# Biliary Cystadenomas: A Case for Complete Resection

**DOI:** 10.1155/2012/501705

**Published:** 2012-06-20

**Authors:** Sastha Ahanatha Pillai, Vimalraj Velayutham, Senthilkumar Perumal, Srinivasan Ulagendra Perumal, Anand Lakshmanan, Sukumar Ramaswami, Ravi Ramasamy, Jeswanth Sathyanesan, Ravichandran Palaniappan, Surendran Rajagopal

**Affiliations:** Institute of Surgical Gastroenterology and Liver Transplantation, Government Stanley Medical College, Chennai—600001, India

## Abstract

*Introduction and Objective*. Biliary cystadenoma is a rare benign neoplasm of the liver with less than 200 cases being reported allover the world. We report a series of 13 cases highlighting the radiological findings and problems related to its management. *Materials and Methods*. Records of thirteen patients who underwent surgery for biliary cystadenomas, between March 2006 and October 2011, were reviewed retrospectively. *Results*. Majority of the patients were females (11 out of 13), with a median age of 46 (23–65) years. The most frequent symptom was abdominal pain (92%). Seven patients had presented with history of previous surgery for liver lesions. Five patients had presented with recurrence after partial resection for a suspected hydatid cyst and two after surgery for presumed simple liver cyst. Ten of the 13 patients had complete resection of the cyst with enucleation in 3 patients, 2 of whom in addition required T-tube drainage of the bile duct. There has been no recurrence during the follow-up period ranging from 3 months to 5 years. *Conclusion*. Biliary cystadenoma must be differentiated from other benign cysts. Hepatic resection or cyst enucleation is the recommended treatment option.

## 1. Introduction

 Biliary cystadenomas are rare cystic lesions of the liver. They account for less than 5% of nonparasitic cysts of the liver [1] and occur frequently in middle-aged women. The size varies from 1.5 to 35 cm [2, 3]. These cysts need to be differentiated from other cystic lesions such as simple cysts, hydatid cysts, abscesses, hematomas, and polycystic liver disease.

 Biliary cystadenomas were first described in 1943 [4]; less than 200 cases have been reported till date. They are often benign lesions with a malignant potential [3, 5, 6]. They develop from either an aberrant bile duct or directly from a primitive hepatobiliary stem cell [1, 7, 8]. Majority are intrahepatic (85%) [6, 7, 9–11], fewer are extra hepatic [5–7, 12] and occasionally are seen to arise from the gall bladder [9, 12].

There is difficulty in differentiating a benign from a malignant biliary cystadenoma and hence these lesions should always be resected. One cannot always reliably distinguish a simple cyst or a hydatid cyst from a benign biliary cystadenoma. In such situations, deroofing, marsupialization, or partial resection of the suspected cysts has resulted in a very high rate of recurrence (>90%) [13].

We report a series of 13 cases highlighting the radiological interpretation of biliary cystadenomas, issues on surgical management, and postoperative followup for recurrence.

## 2. Materials and Methods

**Figure 1:**
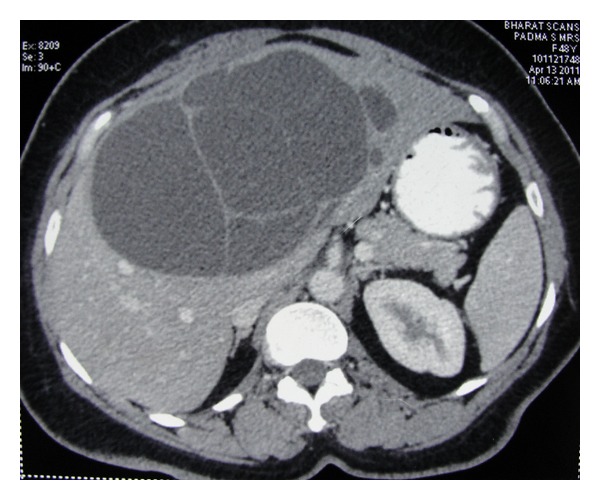
Contrast-enhanced CT scan showing large cystic lesion in liver with multiple-enhancing septae.

Records of patients with histologically confirmed biliary cystadenomas admitted between March 2006 and October 2011 at the Institute of Surgical Gastroenterology and Liver Transplantation, Government Stanley Medical College, Chennai, India, were reviewed retrospectively. Patients' charts were scrutinized for demographic characteristics, clinical presentation, radiological findings, and past and present surgical details and outcomes like morbidity and mortality. Preoperative diagnosis on ultrasound and CECT abdomen was based on presence of one or more findings of internal septations, mural nodules, papillary projections, and cyst wall enhancement (Figure 1). The choice of surgical procedure was based on the site and size of the lesion. Liver resection was the preferred procedure and enucleation was done when resection was not feasible. Postoperative complications and morbidity were recorded. The pathology slides were reviewed. Followup of patients for symptom recurrence was done by telephonic interview. Patients were screened for evidence of recurrence by ultrasound of abdomen. Data were reported as the median with range and percentage.

## 3. Results

Of the 13 patients with biliary cystadenomas, there were eleven women and two men. The median age of the patients was 46 years (range 23–65 years). The most common symptom was abdominal pain (92%), followed by abdominal distension, loss of appetite, and mass abdomen. The median duration of symptoms was 18 months (range 2 to 42 months).

Out of seven patients with history of previous surgery for liver lesions, five patients had partial cystopericystectomies done elsewhere for suspected hydatid cyst, one patient had a laparoscopic marsupialisation and one had laparoscopic fenestration, the latter two for presumed simple cyst of the liver. All seven patients had recurrence of symptoms after a median interval of 4 years (range 2 months to 5 years). Four patients had the postoperative biopsy report as intrahepatic biliary cystadenoma and in three, the histology was inconclusive.

### 3.1. Investigations

Preoperative diagnosis was based on ultrasound and CECT findings. Typical findings included multiloculated cyst with a well-defined capsule, some with wall calcification, internal septations, and solid papillary projections. Three patients had elevated serum gamma glutamyl transferase and serum alkaline phosphatase (Table 1).

### 3.2. Surgery and Outcome

**Figure 2:**
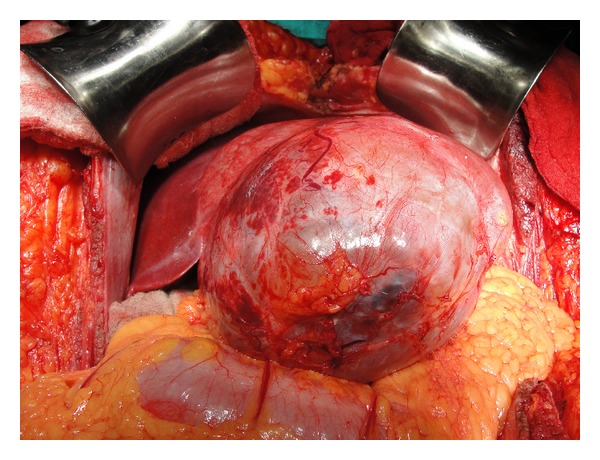
Peroperative picture showing large thick-walled cystic lesion arising from left lobe of liver.

**Figure 3:**
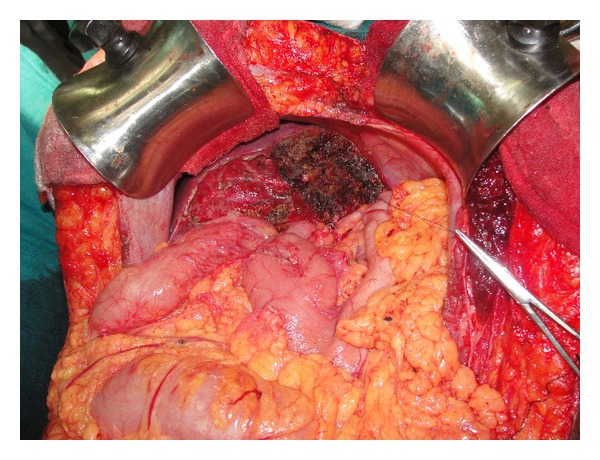
Remnant right lobe of liver after left hepatectomy.

Ten of the 13 patients had resection of the involved segments: four hepatectomies, three bisegmentectomies, two lateral segmentectomies, and one extended right hepatectomy (Figures 2 and 3). In 3 patients, proper hepatic resection could not be performed owing to the large size of the lesion (longest diameter ranging from 23 cm to 34 cm) and the close proximity to the hilum, indenting the hilar structures. These three patients had abdominal pain and distension as the major distressing symptoms. Hence enucleation was contemplated in these patients with a view to relieve the symptoms (Figure 5). Eventually, the histopathology in these three patients turned out to be benign.

T-tube placement was done in 2 of these patients in whom the cyst had a communication with the bile duct. The Preoperative imaging had not demonstrated this biliary communication. A leak test done after enucleation had demonstrated bile leak in the enucleation bed. The site of leak was suture ligated with a T-tube drainage of the common bile duct. The T-tube was removed two weeks later, after confirming absence of a bile leak. Both these patients were free of biliary sequelae or other complications in the followup period of 8 and 14 months.

Intraoperative blood loss ranged from 210–850 mL (median—375 mL). Only one patient required two units of packed red blood cell during surgery. Postoperatively, one patient developed basal atelectasis of the left lung, which settled with supportive care and one patient developed a subdiaphragmatic collection for which ultrasound guided pigtail drainage was done. The median postoperative hospital stay was 14 days (range 10–21).

The postoperative histopathology was intrahepatic biliary cystadenoma in all the patients. There was no evidence of malignancy in any of the patients. None of the patients had recurrence of symptoms or recurrence of the cyst during a median followup of 22 months (range 3 to 66 months). The three patients in whom the lesion was enucleated were specifically followed up with regular USG abdomen even if asymptomatic, which demonstrated no recurrence.

## 4. Discussion

Biliary cystadenomas constitute less than 5% of cystic lesions of the liver. Typically, the patient is a middle-aged woman presenting with abdominal pain and/or discomfort, with distension and a palpable mass [1, 6, 7, 14]. Rare presentations include vomiting, dyspepsia, anorexia, and weight loss [14, 15]. Acute presentation is often pain due to intracystic hemorrhage or rupture of the cyst and fever secondary to infection of the cyst [16]. Jaundice [17–19] is either due to an extrinsic compression of the bile duct [20], biliary obstruction by an intraluminal tumoural mass, or accretion of mucus secretion from a communicating biliary cystadenoma [20]. Ascites is secondary to compression of the inferior vena cava or the hepatic veins [19, 21, 22]. Cystadenomas are known to increase in size during pregnancy and following oral contraceptives suggesting hormonal dependency [1, 2, 13]. Recurrence of a cyst following partial resection should raise a suspicion of cystadenoma.

In our series, abdominal pain was the most common presentation followed by abdominal distension, anorexia and mass abdomen. None of our patients had obstructive jaundice, but three patients had elevated liver enzymes (Table 1).

Biliary cystadenomas are usually large multiloculated cystic tumours and are of two types: those with [1, 5, 8], and those without mesenchymal (ovarian-like) stroma. The ovarian-like stroma is thick consisting of compact spindle-shaped cells and supports the epithelium and is often seen exclusively in women [1, 7, 16]. Microscopically the loculi are limited by single layer of cuboidal or nonciliated columnar epithelium resting on a basement membrane (Figure 6). At places the epithelium forms multiple polypoidal or papillary projections.

Cystadenomas with mesenchymal stroma are considered premalignant with a good prognosis while those without are known to transform to malignancy more often [23] with a poor prognosis [6, 8, 9].

The majority of biliary cystadenomas do not communicate with the bile ducts, but luminal communication may be occasionally observed [2, 24]. In some of the cases, dysplastic mucinous epithelium itself may proliferate within the bile ducts causing obstruction [22]. This variant is considered an intraductal papillary neoplasm with prominent cystic dilatation of the duct rather than a true biliary cystic neoplasm [24, 25]. CT and MRI often fail to identify the narrow communication [2] which is easily demonstratable during an intraoperative cholangiogram [24].

The cystic fluid may be clear and mucinous [5, 7]. Blood stained fluid within the cyst indicates a malignant component (cystadenocarcinoma). Rarely, the fluid may be bile stained, purulent, proteinaceous, or gelatinous [9]. The septa within the cyst may rarely show calcification.

 Differential diagnosis of cystadenomas include simple liver cysts, parasitic cysts (particularly hydatid cyst), haematomas, post-traumatic cysts, liver abscess, polycystic diseases biliary cystadenocarcinoma, and metastatic, ovarian, or pancreatic adenocarcinoma [9, 10, 14]. Extra hepatic biliary cystadenomas can typically mimic choledochal cyst [26].

**Figure 4:**
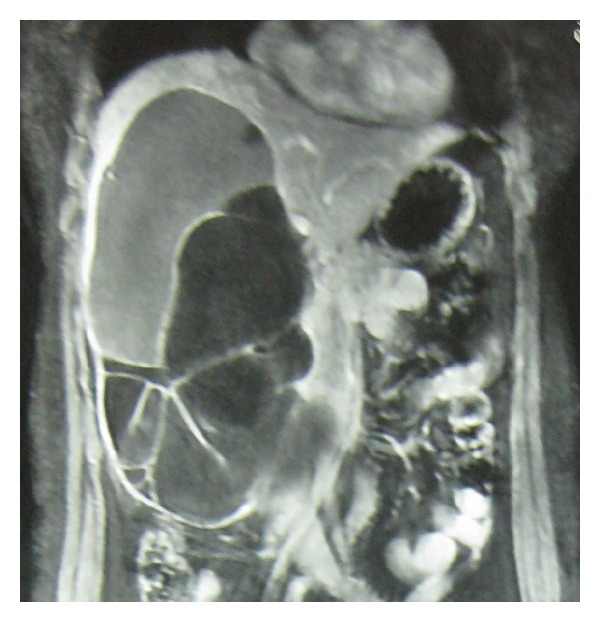
T1-weighted MRI image showing large multiseptated cystic lesion in right lobe of liver.

**Figure 5:**
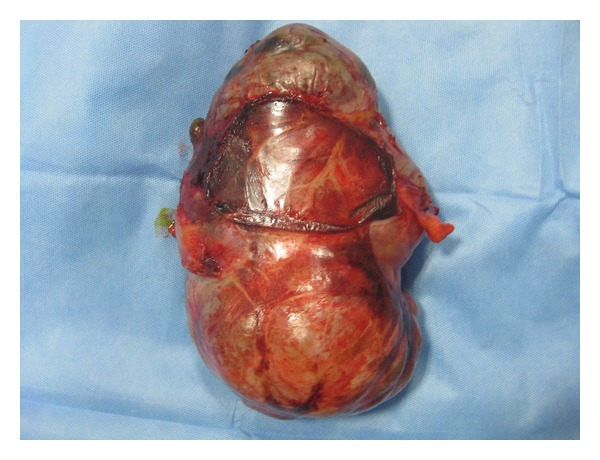
Enucleated specimen of biliary cystadenoma.

**Figure 6:**
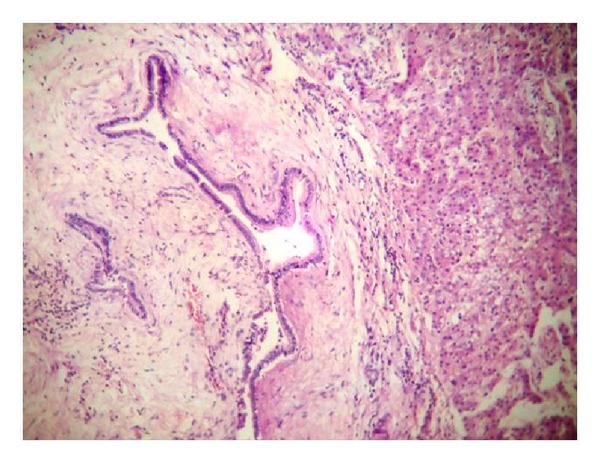
10× histology picture showing dilated bile duct with low columnar epithelium and spindle-shaped cells in stroma (ovarian like stroma).

Diagnosis of biliary cystadenomas is often possible on an ultrasonography, CECT, and MRI abdomen (Figure 4). On ultrasound, cystic nature of the lesion with multiple loculi, septations, and internal echoes, with papillary projections [9, 15], is typical. The cyst is well-demarcated and thick-walled, globular or ovoid with a noncalcified wall [7–9, 27]. Doppler study may show the vascular flow within the lesion [28]. Dilation of the biliary system can also be seen. CECT in addition demarcates the anatomic relation to surrounding structures, particularly major vessels [14, 15, 28, 29]. Coarse calcifications in the septae [28, 30] may be seen. MRI is able to characterise the nature of fluid within the cyst [9, 28], that is, blood versus mucin. Despite all the aforementioned radiological features, the Preoperative radiological diagnostic accuracy may be as low as 30% and therefore a high index of suspicion is indicated [31]. An irregular thickness of the cyst wall, presence of mural nodules, or papillary projections indicates the possibility of a malignancy [9, 10, 28, 32]. Hypervascularity of mural nodules on CT also suggests malignancy [33].

In our series, CT had shown multiple loculi and septations in all patients. Well-defined capsule was seen in eight patients (61%) and wall-enhancement in five patients (38%). Papillary projections and calcifications were seen in three patients each.

A Preoperative core needle biopsy to detect malignancy is not recommended as this is not accurate and carries the risk of needle seeding and dissemination [16, 34, 35]. Elevated CEA and CA 19-9 in the serum or the cystic fluid aids in diagnosis and followup of patients [16, 36]. A normal level does not exclude a biliary cystadenoma; some simple liver cysts may also show elevated serum or cystic fluid CEA or CA 19-9 [36].

Two issues are of importance while making a diagnosis of biliary cystadenoma. One is the incomplete excision of the cyst, misdiagnosed as a simple cyst or a hydatid cyst, resulting in recurrence and the second is the difficulty in differentiating biliary cystadenomas from biliary cystadenocarcinomas [1, 5, 9, 12], either before or during surgery. Hence the recommendation is a complete resection of any suspected biliary cystadenoma [1, 5, 12]. In our series, five patients presented to us with recurrence after partial resection done elsewhere for a mistaken diagnosis of hydatid cyst and two patients with recurrence after surgery for a suspected simple liver cyst.

Earlier, biliary cystadenomas have been treated with various procedures like marsupialization, internal Roux-en-Y drainage, aspiration, sclerosis, or partial resection. However, all these procedures have been associated with high rates of recurrence [7, 9, 14, 37, 38]. Hence, complete resection is the treatment of choice with negligible recurrence [8]. Pinson et al. [39] have reported cyst enucleation without late recurrence and mortality. This procedure is a valid alternative where resection is difficult or is likely to be associated with morbidity [12, 16].

In conclusion, the diagnosis of biliary cystadenoma should be considered in any multilocular cystic lesion of the liver, particularly in a middle-aged woman. It is an important differential diagnosis for a hydatid cyst especially in endemic regions and a complete resection of the cyst is recommended when in doubt. The recommended treatment of choice for any suspected biliary cystadenoma is resection as it is extremely difficult to differentiate Preoperatively, a benign from a malignant neoplasm. Enucleation is another option and is indicated where resection is impossible due to the size and location of the tumour.

## Figures and Tables

**Table 1:** Clinical and radiological characteristics.

Clinical features	
Abdominal pain	12 (92%)
Abdominal distension	5 (38%)
Loss of appetite	5 (38%)
Mass abdomen	4 (31%)
History of previous surgery	7 (54%)
Elevated LFT	3 (23%)

Radiological features on CECT	

Multiloculated cyst	13 (100%)
Internal septations	13 (100%)
Well defined capsule	8 (61%)
Enhancing cyst wall	5 (38%)
Papillary projections	3 (23%)
Calcification	3 (23%)
